# Autophagy and Inflammasome Activation in Dilated Cardiomyopathy

**DOI:** 10.3390/jcm8101519

**Published:** 2019-09-21

**Authors:** Angela Caragnano, Aneta Aleksova, Michela Bulfoni, Celeste Cervellin, Irene Giulia Rolle, Claudia Veneziano, Arianna Barchiesi, Maria Chiara Mimmi, Carlo Vascotto, Nicoletta Finato, Sandro Sponga, Ugolino Livi, Miriam Isola, Carla Di Loreto, Rossana Bussani, Gianfranco Sinagra, Daniela Cesselli, Antonio Paolo Beltrami

**Affiliations:** 1 Department of Medicine, University of Udine, 33100 Udine, Italy; angelacaragnano@alice.it (A.C.); michela.bulfoni@uniud.it (M.B.); celestecervellin@gmail.com (C.C.); irenegiulia.rolle@gmail.com (I.G.R.); claudia.veneziano@uniud.it (C.V.); barchiesi.arianna@gmail.com (A.B.); chiara.mimmi@gmail.com (M.C.M.); carlo.vascotto@uniud.it (C.V.); nicoletta.finato@uniud.it (N.F.); ugolino.livi@uniud.it (U.L.); miriam.isola@uniud.it (M.I.); carla.diloreto@uniud.it (C.D.L.); 2 Cardiovascular Department, Azienda Sanitaria Universitaria di Trieste and Department of Medical Surgical and Health Sciences, University of Trieste, 34100 Trieste, Italy; aaleksova@gmail.com (A.A.); gianfranco.sinagra@asuits.sanita.fvg.it (G.S.); 3 Centre of New Technologies, University of Warsaw, 02-097 Warsaw, Poland; 4 Cardiothoracic Department, Azienda Sanitaria Universitaria Integrata di Udine, 33100 Udine, Italy; sandro_sponga@yahoo.com; 5 Institute of Pathological Anatomy, Azienda Sanitaria Universitaria di Trieste and Department of Medical Surgical and Health Sciences, University of Trieste, 34100 Trieste, Italy

**Keywords:** Dilated cardiomyopathy, inflammasome, interleukin 1 β, autophagy, branched chain amino acids, mechanistic target of rapamycin

## Abstract

Background: The clinical outcome of patients affected by dilated cardiomyopathy (DCM) is heterogeneous, since its pathophysiology is only partially understood. Interleukin 1β levels could predict the mortality and necessity of cardiac transplantation of DCM patients. Objective: To investigate mechanisms triggering sterile inflammation in dilated cardiomyopathy (DCM). Methods: Hearts explanted from 62 DCM patients were compared with 30 controls, employing immunohistochemistry, cellular and molecular biology, as well as metabolomics studies. Results: Although misfolded protein accumulation and aggresome formation characterize DCM hearts, aggresomes failed to trigger the autophagy lysosomal pathway (ALP), with consequent accumulation of both p62^SQSTM1^ and dysfunctional mitochondria. In line, DCM hearts are characterized by accumulation of lipoperoxidation products and activation of both redox responsive pathways and inflammasome. Consistently with the fact that mTOR signaling may impair ALP, we observed, an increase in DCM activation, together with a reduction in the nuclear localization of Transcription Factor EB -TFEB- (a master regulator of lysosomal biogenesis). These alterations were coupled with metabolomic alterations, including accumulation of branched chain amino acids (BCAAs), known mTOR activators. Consistently, reduced levels of PP2Cm, a phosphatase that regulates the key catabolic step of BCAAs, coupled with increased levels of miR-22, a regulator of PP2Cm levels that triggers senescence, characterize DCM hearts. The same molecular defects were present in clinically relevant cells isolated from DCM hearts, but they could be reverted by downregulating miR-22. Conclusion: We identified, in human DCM, a complex series of events whose key players are miR-22, PP2Cm, BCAA, mTOR, and ALP, linking loss of proteostasis with inflammasome activation. These potential therapeutic targets deserve to be further investigated.

## 1. Introduction

Dilated cardiomyopathy (DCM) is a syndrome characterized by cardiac enlargement and impaired systolic function, with an ejection fraction <45% [1]. Although in 35%–40% of cases DCM is idiopathic (i.e., detectable causes can be excluded), the screening of first-degree relatives revealed a positive family history in 20%–35% of cases, suggesting a genetic etiology [2]. Pathogenetic mutations have been detected in >40 genes [3], but truncating mutations of the *titin* gene occur in about 25% of familial cases and in 18% of sporadic cases [4]. Nonetheless, a genetic cause could be identified in just 30%–35% of familial DCM, and the frequencies of pathogenetic mutations in any single gene ranges from 1% to 25% [2,4].

Genetic DCM can result from mutations affecting cytoskeletal and sarcomeric proteins that may perturb force generation [3]. However, the genotype/phenotype relationship is not straightforward, since mutations in the same gene may have completely different clinical outcomes [5]. This heterogeneity may be explained by disruption of specific protein binding partners, the presence of comorbidities, differences in penetrance, or genetic resistance to adverse remodeling [2]. However, the pathophysiologic mechanism of genetic DCM may be much more complex, involving the finely tuned mechanism of protein synthesis and degradation (i.e., proteostasis) that maintains cell homeostasis [6]. Specifically, the expression and degradation of a mutant or misfolded protein are regulated by nonsense-mediated mRNA decay [7,8], molecular chaperones [9], the endoplasmic reticulum assisted degradation (ERAD) via the ubiquitin–proteasome system (UPS) [10], and the autophagy–lysosomal pathway (ALP), including chaperone-assisted selective autophagy (CASA) [9], to minimize the levels of toxic proteins or protein aggregates. The emerging role of proteostasis in the pathogenesis of DCM is shown by the accumulation of amyloid-like substances and polyubiquitinated proteins in patients affected by DCM [11,12]. Whether UPS and ALP impairment are involved in the pathogenesis of DCM in humans is less clear [6]. Intriguingly, in conditions of UPS overload, aggregation-prone proteins are ubiquitinated and segregated by the cells in perinuclear locations, close to the microtubule-organizing centers, where they are encaged within a net of intermediate filaments (e.g., keratins or vimentin) that colocalize with histone deacetylase 6 [13]. The latter promotes autophagosome maturation and macro-autophagy (here referred to as autophagy) [13]. Autophagy is a highly regulated process that promotes the isolation of long-lived cytosolic proteins, organelles, and part of the cytoplasm within a transient double membrane (i.e., the phagophore), that expands and closes to become an autophagosome, which eventually fuses with the lysosome to promote cargo degradation [14]. The unc-51-like autophagy-activating kinase (Ulk) macromolecular complex promotes initiation of the isolation membrane by activating the Vps34 complex (which comprises Beclin1). The latter complex plays a critical role in autophagosome expansion, through the formation of phosphatidyl inositol 3-phosphate (PI3P). Indeed, PI3P recruits several Atg proteins, including Atg18, Atg20, Atg21, and Atg24, promoting phagophore expansion. Maturation and closure of the autophagosome are regulated by the Atg12–Atg5 complex (whose formation is mediated by Atg3 and Atg7), that eventually leads to the conjugation of microtubule-associated protein 1 light chain 3 (LC3) to phosphatidylethanolamine [14].

However, when the ALP is impaired, dysfunctional mitochondria can accumulate in cells, triggering the activation of the inflammasome and the release of inflammatory cytokines, such as IL1β [15]. Importantly, we recently demonstrated that IL1β levels can predict long-term mortality and necessity for cardiac transplantation in a cohort of ambulatory patients affected by DCM [16].

In this study, we verified if loss of proteostasis and ALP impairment, coupled with the activation of the inflammasome, could be associated with the release of proinflammatory cytokines and account for the clinical heterogeneity of patients affected by idiopathic DCM. Furthermore, we evaluated the potential therapeutic impact of downregulating miR-22, an inhibitor of ALP that could be responsible for the metabolic derangements, accumulation of biologically active metabolites, and increased senescence rate of cardiac pericytes that characterize DCM hearts.

## 2. Experimental Section

### 2.1. Patient Enrollment and Ethics

DCM patients transplanted at our institution were enrolled, and hearts were analyzed by an expert pathologist, as in [17]. Hearts of patients that died for causes other than cardiovascular disease were used as controls. Histological studies were conducted on 50 patients and 18 controls, while the metabolomics study was conducted on another 12 patients and 12 controls. Table S1 summarizes the clinical features of patients and controls. Western blotting studies, shown in supplementary Figure S1, were conducted on 7 patients and 7 autoptic controls, whose characteristics are summarized in Table S1. The study, conducted in accordance with the Declaration of Helsinki, was approved by the Ethics Committee of Udine (2 August 2011, ref. 47831) and by the Internal Review Board of the University of Udine (29 August 2017, ref. 10/IRB DAME_BELTRAMI_17). At time of cardiac transplantation, signed informed consent was collected from patients enrolled in the study.

### 2.2. Tissue Sampling

Explanted hearts were sampled as in [17], fixed in 10% buffered formalin, and embedded in paraffin (FFPE). For molecular and metabolomics analyses, samples obtained both from atria and ventricles were snap frozen in liquid nitrogen.

### 2.3. Histology, Histochemistry, Immunohistochemistry, and Immunofluorescence Assays

Tissue sections (5 μm thick) were cut and processed for further histochemical, immunohistochemistry, and immunofluorescence analyses. Gomori trichrome staining was performed for fibrosis assessment, while Congo red staining was employed to detect amyloids. Immunohistochemistry and immunofluorescence labeling were performed as indicated in Table S2. Images were acquired employing either a transmitted light microscope (Leica DMD 108, Leica, Wetzlar, Germany), a confocal microscope (Leica TCS-SP2 or Leica TCS-SP8), or an epifluorescence microscope (Leica DMI 6000B). Morphometric analyses were carried out by employing ImageJ software (https://imagej.net/Welcome).

### 2.4. Western Blotting

Frozen cardiac samples were homogenized in Radioimmunoprecipitation assay buffer (RIPA) buffer added with protease inhibitors (all from Sigma-Aldrich, St. Louis, MI, USA). Protein extracts were stored at −80 °C until they were analyzed. Antibodies are indicated in Table S2.

### 2.5. NMR Analysis

Frozen samples were homogenized, dried, dissolved in H_2_O/MeOH (1/20), and stored at −80 °C before NMR analysis. Dry extracts were re-dissolved in D_2_O phosphate buffer, pH 7.4, and NMR spectra were acquired on a Bruker Avance 500 MHz spectrometer (Bruker BioSpin, Rheinstetten, Germany). A data matrix of 24 samples and 451 spectra buckets, or NMR features, was uploaded to the MetaboAnalyst 3.0 platform [18] for statistical analysis with R v3.2.2 (http://www.R-project.org). Each bucket was treated as an independent variable of both control and DCM groups and analyzed with an unpaired *t*-test. Hierarchical clustering was performed with the “hclust” function of “stat” R package on normalized data after autoscale feature standardization. Pearson’s correlation was used for distance measurement parameters, and Ward’s linkage was used as the clustering algorithm. Concentration tables for 22 identified metabolites were uploaded on the Metabo-Analyst 3.0 platform for pathway enrichment analysis and analyzed with the global test approach [19]. Pathway topology analysis was performed by a relative betweenness centrality approach [20].

### 2.6. Cardiac Pericyte/Mural Cell Culture and in Vitro Characterization

Cardiac pericytes (CPc) were cultured from atrial biopsies collected at the time of cardiac transplantation, as in [21]. Immunofluorescence assays were conducted on 4% paraformaldehyde-fixed cells employing antibodies and protocols indicated in Table S2.

### 2.7. Oxygen Consumption Rate (OCR)

OCR was determined by direct measurement with a SeaHorse Extracellular Flux Analyzer XpE instrument (Seahorse Bioscience, Agilent Technologies, Santa Clara, CA, USA). OCR for the mitochondrial stress test was determined following the manufacturer’s instructions. For statistical analyses, all OCR values were normalized with those of one control CPc line, then the measurements of the four control CPc lines were averaged, and the same was done with the four CPc lines obtained from the DCM patients.

### 2.8. RNA Extraction and Real-Time PCR Analysis of miRNA Expression

Total RNA was extracted from CPc using the mirVana™ miRNA Isolation kit (Ambion, Thermo Fisher, Waltham, MA, USA) and from DCM and control FFPE tissues using the RecoverAll™ (Thermo Fisher). Total Nucleic Acid Isolation kit (Ambion, Thermo Fisher) was used following the manufacturer’s instructions. miRNA reverse transcription was performed using the TaqMan MicroRNA Reverse Transcription Kit (Applied Biosystems), using specific primers. A total of 1.3 L of the RT product were used for the real-time qPCR assay, employing a master mix (TaqMan Universal PCR Master Mix, with no UNG, Applied Biosystems) and TaqMan probes to evaluate the expression profiles of miR-22, miR-146a, and miR-146b. miR-16 and miR-92 were employed as endogenous controls for cells and tissues, respectively. The amplification protocol was carried out using the LightCycler 480 (Roche, Basel, Switzerland) instrument.

### 2.9. MiRNA Inhibition

CPc were transfected at 70% confluence with either 50 nM anti-hsa-miR-22-3p or with 50 nM Negative Control#1 (mirVana miRNA Inhibitor 2.0, Life technologies, Carlsbad, CA, USA) employing lipofectamine diluted in Opti-MEM (Gibco, Dublin, Ireland), following the manufacturer’s instructions. Forty-eight hours after transfection, cells were fixed with 4% paraformaldehyde.

### 2.10. Statistical Analysis

Gaussian distribution was assessed by Kolmogorov–Smirnov tests. Characteristics of the study population were described using mean ± SD or median (10th and 90th percentiles), as appropriate. Continuous variables between two groups were compared using *t*-tests or Mann–Whitney tests, as appropriate. Comparisons of continuous variables among groups were performed by ANOVA followed by Bonferroni post-test or by Kruskal–Wallis followed by Dunn’s post-test, as appropriate. Correlations between two variables were analyzed employing Pearson or Spearman tests, as appropriate. *p* values < 0.05 were considered significant. Analyses were conducted with Prism, version 4.0c and SPSS20 for Macintosh software.

## 3. Results

### 3.1. Failing Hearts of DCM Patients Show Accumulation of Misfolded and Ubiquitinated Proteins

Ventricular biopsies of transplanted patients (*n* = 50) were compared with biopsies of control hearts (*n* = 18). Failing hearts were severely dilated and functionally impaired (Table 1).

**Figure 1:**
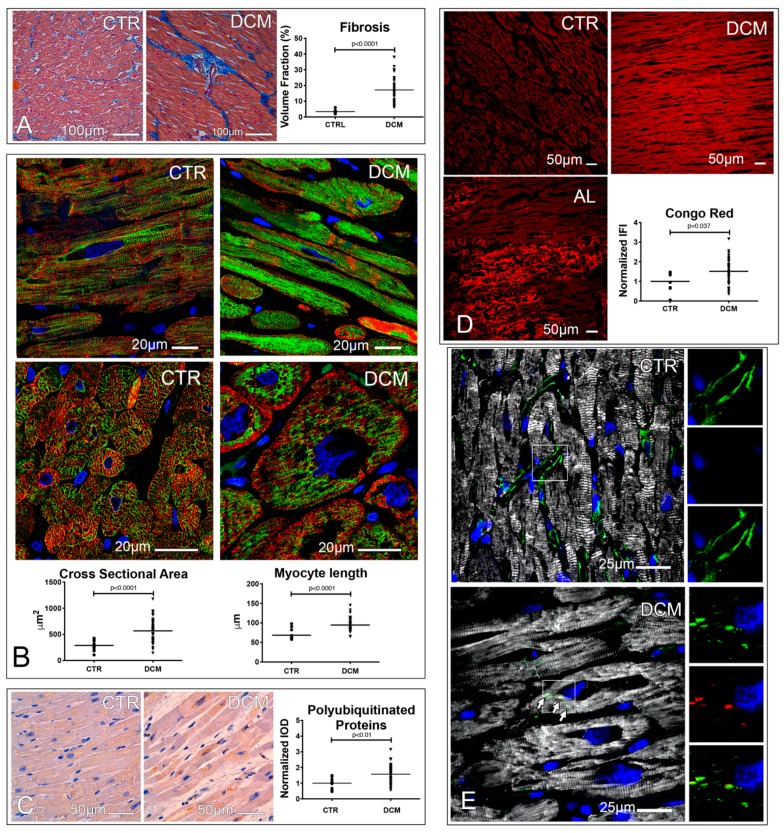
Fibrosis, hypertrophy, and loss of proteostasis characterize the hearts of dilated cardiomyopathy (DCM) patients. (**A**) Fibrosis. Microimages of trichrome stained control and diseased hearts, showing interstitial and perivascular fibrosis (blue). Dot plots indicate the volume fraction occupied by fibrosis; an unpaired *t* test was employed. (**B**) Hypertrophy. Confocal images of cardiac sections showing α-sarcomeric actin (ASA, red) and desmin (green) stained cardiomyocytes. Cardiomyocyte cross-sectional area and length are shown in dot plots; unpaired *t* test and Mann–Whitney test, respectively, were employed. (**C**–**E**) Protein misfolding. Microimages of polyubiquitinated protein labeled cardiac sections (brown). Normalized integrated optical density (IOD) values, obtained dividing individual IOD values by the mean IOD of controls, of the immunohistochemistry staining are shown in dot plots; an unpaired *t* test was employed (**C**). Confocal images of Congo red (CR) stained cardiac sections of control, DCM, and AL amyloidosis (AL) patients. CR was excited with a UV laser, while the fluorescence emission range was set at 596–615 nm employing a monochromator. The integrated fluorescence intensity (IFI) values of CR, normalized as IOD, are shown in dot plots; a Mann–Whitney test was employed (**D**). Confocal images showing the presence of aggresomes. ASA (white) labels cardiomyocytes, while vimentin decorates both interstitial cells and aggresomes (green), these latter being characterized by the co-localization of vimentin and p62^SQSTM1^ (red). Images of either vimentin, p62^SQSTM1^, or the combination of the two stainings without ASA are shown in the right portion of each figure (**E**). Nuclei of fluorescence images are stained in blue by 4′,6-diamidine-2′-phenylindole dihydrochloride (DAPI). A total of 18 controls and 50 DCM hearts were analyzed.

DCM hearts displayed significant degrees of cardiomyocyte fibrosis, hypertrophy, and accumulation of polyubiquitinated proteins (Figure 1A–C). Protein quality control failure and intracellular protein fibrillation were shown by the positivity of DCM hearts to Congo red staining (Figure 1D). In DCM myocytes, but not in control myocytes, some misfolded proteins were sequestered by the aggresome (i.e., cytoplasmic inclusions containing ubiquitinated proteins encaged in a net of vimentin or keratin filaments; Figure 1E) [13].

Therefore, failing DCM hearts are characterized by loss of proteostasis, accumulation of amyloid-like substances, and the formation of aggresomes in cardiomyocyte cytoplasm.

### 3.2. A Defective Autophagy Lysosomal Pathway Characterizes Failing DCM Hearts

**Figure 2:**
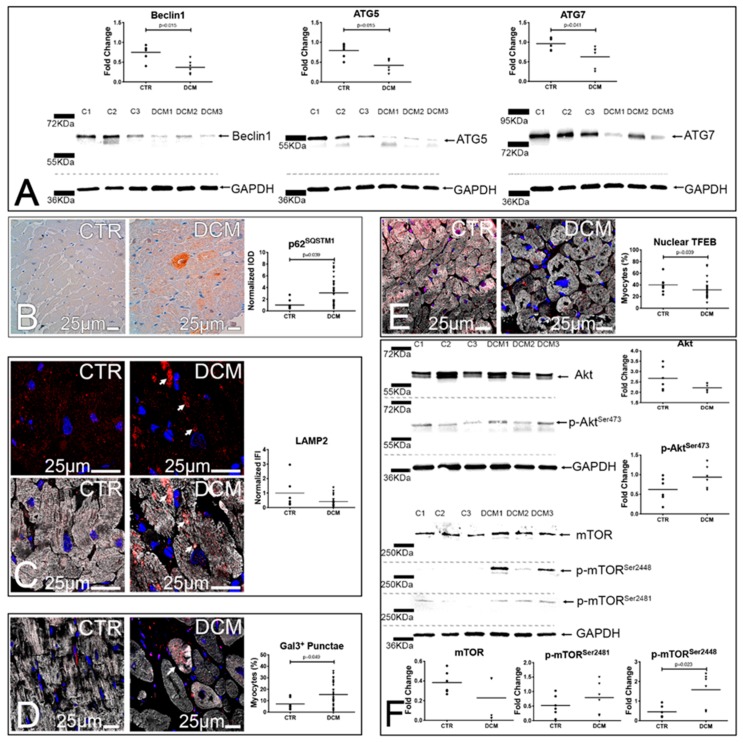
Defects of the autophagy lysosomal pathway characterize the hearts of DCM patients. (**A**,**B**) Autophagy. Western blots (WBs) of whole protein extracts of 3 control hearts and 3 DCM explanted hearts. Blotted proteins were incubated with antibodies against Beclin 1, Atg5, Atg7, and GAPDH. Densitometric analyses of 6 DCM and 6 controls are shown in dot plots; unpaired *t* tests (Beclin 1 and Atg 5) and Mann–Whitney tests (Atg 7) were employed (**A**). Microimages of p62^SQSTM1^-labeled cardiac sections (brown, (**B)**). Quantitative analysis of p62^SQSTM1^ staining is shown in dot plots; a Mann–Whitney test was employed. (**C**–**E**) Lysosome dysfunction and biogenesis. Confocal images of LAMP2- (red) and ASA-labeled (white) cardiac sections. Arrows point to enlarged lysosomes. Normalized IFI values of LAMP2 staining are shown in dot plots; a Mann–Whitney test was employed (**C**). Confocal images of Galectin3- (red) and ASA-labeled (white) cardiac sections. Arrow points to a myocyte with Galectin3 punctae. Dot plots display the fraction of cardiomyocytes showing Galectin3 punctae in their cytoplasm; an unpaired *t* test was employed (**D**). Confocal images of TFEB- (red) and ASA-stained (white) cardiac sections. Box plots illustrate the fraction of cardiomyocytes showing TFEB nuclear positivity; a Mann–Whitney test was employed (**E**). In fluorescence images, DAPI labels nuclei (blue). (**F**) mTOR activation status. WBs of whole protein extracts of 3 control hearts and 3 DCM explanted hearts. Blotted proteins were incubated with antibodies against Akt, p-Akt^Ser473^, mTOR, p-mTOR^Ser2448^, p-mTOR^Ser2481^, and GAPDH. Densitometric analyses of 6 DCM and 6 controls are shown in dot plots; unpaired *t* tests (Akt, p-Akt^Ser473^, p-mTOR^Ser2481^ and p-mTOR^Ser2448^) and Mann–Whitney tests (mTOR), as appropriate, were employed. For histology, 18 controls and 50 DCM hearts were analyzed.

Although aggresome formation should promote the removal of unfolded proteins via the ALP (aggrephagy) [13], DCM hearts were characterized by a significantly reduced expression of Beclin 1, Atg5, and Atg7 (Figure 2A) and by a significant accumulation of p62^SQSTM1^, suggesting a discrepancy between aggresome formation and the ability of DCM hearts to remove aggregates (Figure 2B). In line, lysosomes in DCM hearts were characterized by rarefaction, pathologic enlargement, and lysosomal membrane permeabilization (LMP), as demonstrated by galectin 3 punctae formation [22] (Figure 2C–D). Consistently, nuclear TFEB, a master regulator of the coordinated lysosomal expression and regulation) (CLEAR) gene network [23], involved in lysosomal biogenesis and function, was significantly reduced in both DCM cardiomyocytes and interstitial cells (Figure 2E and Figure S2). Since mTOR activation could account for the observed results, we analyzed the Akt/mTOR axis, and we observed a trend towards higher levels of phosphoAkt^Ser473^ (*p* = 0.099), associated with a significant increase in the levels of mTOR phosphorylated in Ser2448, and a trend towards higher levels of mTOR phosphorylation in Ser2481 (Figure 2F). The activation status of mTOR was further confirmed by assessing the levels of 4EBP-1, a target of mTORC1 complex, phosphorylated on threonins 37 and 46. DCM hearts were characterized, with respect to autoptic hearts obtained from patients that died for causes other than cardiac disease, by increased levels of 4EBP-1 phosphorylation (Figure S1).

These results indicate that, in DCM, mTOR activation is associated with ALP suppression and accumulation of proteinaceous aggregates in the form of aggresomes.

### 3.3. Accumulation of Dysfunctional Mitochondria, Oxidative Stress and Inflammasome Activation

**Figure 3:**
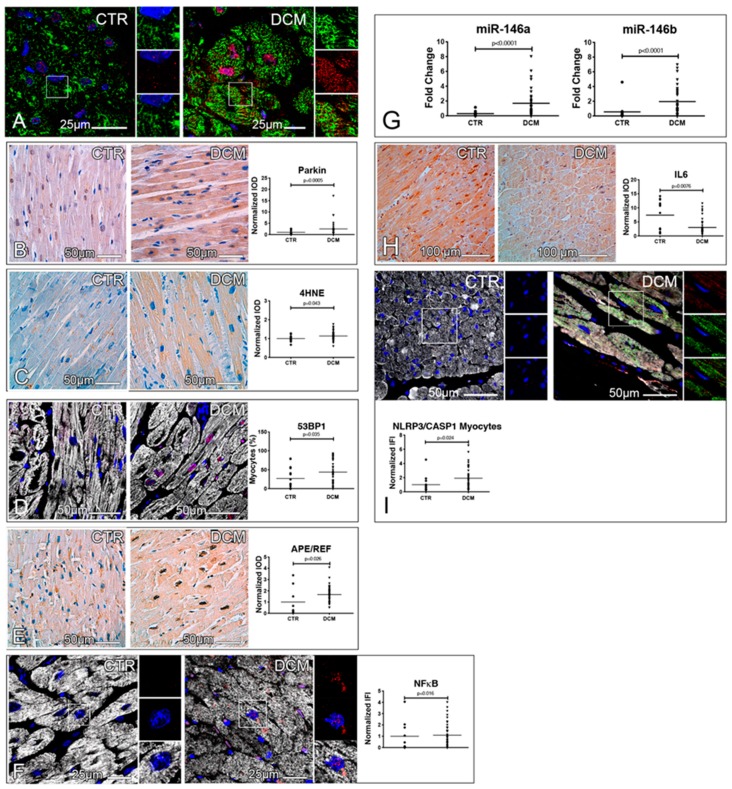
Evidence of the accumulation of dysfunctional mitochondria, lipoperoxidation products, and activated inflammasomes in explanted hearts. (**A**,**B**) Mitochondrial dysfunction. Confocal images of cardiac sections immunostained for mitochondria (green) and Parkin (red). Higher magnifications of the insets are shown, as single channel images of mitochondria and Parkin, in the right portion of each figure (**A**). Microimages of Parkin immunohistochemistry stainings of cardiac sections (brown). Normalized IOD values of Parkin are shown in dot plots; a Mann–Whitney test was employed (**B**). (**C**–**E**) Oxidative stress and redox signaling. Microimages of 4-hydroxynonenal (4HNE)-labeled cardiac sections (brown). Normalized IOD values of 4HNE are shown in dot plots; an unpaired *t* test was employed (**C**). Confocal images of cardiac sections immunostained for 53BP1 (red) and ASA (white). The fraction of cardiomyocytes showing 53BP1 nuclear positivity is shown in dot plots; a Mann–Whitney test was employed (**D**). APE/REF immunohistochemistry staining of cardiac sections (brown). Results of the quantitative assessment of the nuclear expression of APE/REF are shown in the right panel as normalized IOD; a Mann–Whitney test was employed (**E**). (**F**–**I**) Inflammatory pathways. Confocal images of p65 NFκB- (red) and ASA-labeled (white) cardiac sections. Higher magnifications of the insets are shown in the right portion of each figure. Normalized IFI values of NFκB staining are shown in plots (**F**). Dot plots showing the expression of miR-146a and miR-146b in controls and DCM hearts, as assessed by RT-PCR. Data were normalized employing the 2^−ΔΔCt^ method, using *miR-92a* as housekeeping gene; Mann–Whitney tests were employed (**G**). IL6 immunohistochemistry staining of cardiac sections (brown). Normalized IOD values of IL6 staining are shown in dot plots; a Mann–Whitney test was employed (**H**). Epifluorescence images of NLRP3- (green), caspase1- (red), and ASA-labeled (white) cardiac sections. Higher magnifications of the insets are shown in the right portion of each figure. Normalized IFI values of caspase1 staining colocalized with NLRP3 positivity are shown in dot plots; a Mann–Whitney test was employed (**I**). In fluorescence images, DAPI-labeled nuclei are shown in blue. A total of 18 controls and 50 DCM hearts were analyzed for histology and RT-PCR experiments.

**Figure 4:**
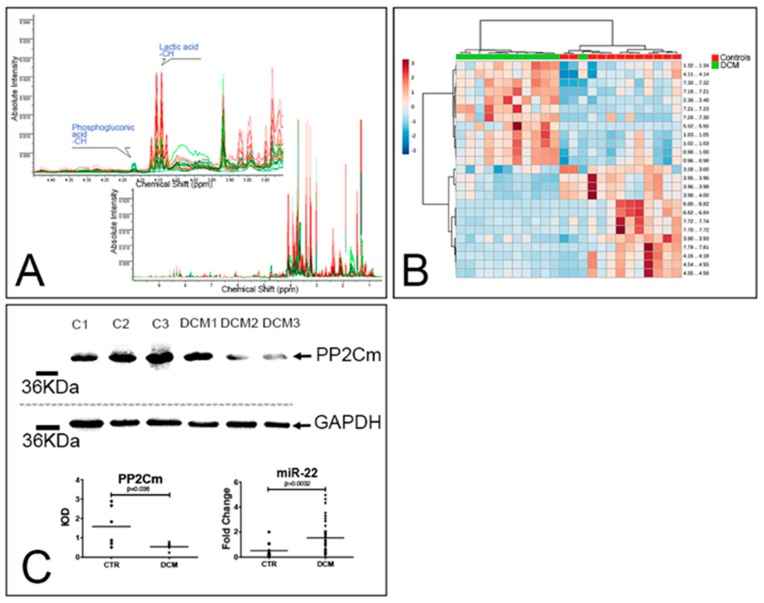
DCM is associated with alterations of amino acid metabolism. (**A**,**B**) Metabolomic analysis. 1D NMR spectra of 12 control (green lines) and 12 DCM hearts (red lines). The full spectra are shown in the bottom panel, while the top panel shows the 3.75–4.45 ppm region and the peaks assigned to lactic acid and 6-phosphogluconic acid (**A**). Heatmap summarizing the results of the hierarchical clustering analysis generated employing the top 25 features ranked by *t* tests. Each colored cell on the map corresponds to a concentration value in the data table, with samples in columns and features/compounds in rows (**B**). (**C**) WB and real-time PCR analyses. Representative WB of whole-protein extracts of 3 control hearts and 3 DCM explanted hearts. Blotted proteins were incubated with antibodies against PP2Cm and GAPDH. Results of the densitometric analyses (*n* = 6 controls and 6 DCM) are reported in the bottom-left panel; a Mann–Whitney test was employed. The bottom-right panel summarizes the results of the RT-PCR analysis of miR-22 expression in control (*n =* 12) and DCM (*n =* 37) hearts; a Mann–Whitney test was employed. Fold change was computed using *miR-92a* as a housekeeping gene.

ALP is crucial to remove dysfunctional mitochondria [24]. Consistently, dysfunctional mitochondria positive for Parkin, an E3 ubiquitin ligase stabilized on depolarized mitochondrial membranes, were detected in DCM cardiomyocytes (Figure 3A). Moreover, the cytoplasmic levels of both Parkin (Figure 3B) and 4-hydroxynonenal (4HNE), a product of lipoperoxidation of the mitochondrion-specific phospholipid cardiolipin [25], were significantly incremented in DCM (Figure 3C). Western blot (WB) analysis of Parkin expression, assessed in DCM hearts and autoptic controls, corroborated these results (Figure S1). These findings were paralleled by both an incremented frequency of cardiomyocytes and interstitial cells positive for the DNA damage checkpoint protein 53BP1 (Figure 3D and Figure S2), and an increased expression and nuclear localization of the protein apurinic/apyrimidinic endonuclease Ref-1-protein (APE/Ref-1, Figure 3E), a multifunctional protein regulating NFκB in a redox-dependent fashion. Consistently, DCM hearts were characterized by a significant increase of both NFκB levels and two NFκB target gene products (i.e., miR-146a and miR-146b), which exert negative feedback on the axis (Figure 3F–G and Figure S2) [26]. In line, IL6 levels were significantly downregulated in DCM hearts (Figure 3H).

Next, we studied the activation status of the inflammasome, a multiprotein platform, activated by damage-associated molecular patterns (DAMPs) via pattern-recognition receptors (PRR), responsible for caspase1 activation that, in turn, processes and promotes the secretion of IL1δ. The levels of caspase1 co-localized with the PRR NLRP3 were significantly increased in DCM cardiomyocytes (Figure 3I).

Last, we evaluated whether clinical data in DCM patients were associated with the analyzed parameters. Indeed, we observed that the duration of the disease was inversely correlated with the expression of nuclear TFEB in cardiomyocytes (p = −0.415, *p* = 0.049), while left ventricular ejection fraction positively correlated with the fraction of interstitial cells expressing nuclear TFEB (*p* = 0.45, *p* = 0.021). Importantly, p62^SQSTM1^ levels positively correlated with the fraction of 53BP1^+^ senescent myocytes and interstitial cells (*p* = 0.45, *p* < 0.01; *p* = 0.37, *p* = 0.03, respectively), NFpB levels in non-myocytes (*p* = 0.58, p < 0.01), and the levels of co-localized NLRP3/caspase1 in myocytes (*p* =0.56, *p* < 0.01). Moreover, in cardiomyocytes, Parkin levels positively correlated with NFκB levels (*p* =0.34, *p* = 0.027).

In summary, in DCM, autophagic arrest and p62^SQSTM1^ accumulation, coupled with mitochondrial dysfunction, are associated with inflammasome activation. Additionally, lysosomal dysfunction correlates with both disease duration and systolic dysfunction.

### 3.4. Metabolic Alterations in DCM Hearts

Next, we evaluated if mitochondrial dysfunction was coupled with alterations of cardiac metabolism, as occurs in a mouse model of DCM that evolves to heart failure [27].

1D ^1^H-NMR and 2D ^1^H-^13^C-HMQC and ^1^H-^1^H-TOCSY spectra of hydrophilic metabolites extracted from myocardial samples of DCM patients and controls (*n* = 12 each; Table 2) were acquired.

A total of 451 spectra buckets (i.e., NMR features) were detected (Figure 4A). By employing the top 25 buckets, evidenced by univariate statistical analysis, hierarchical clustering analysis correctly discriminated DCM from controls (Figure 4B). Within the 22 metabolites that were unambiguously identified by 1D NMR, 10 compounds were significantly affected by pathology (Table S3). Lactate, pyruvate, and fumarate, as well as the amino acids valine, leucine, phenylalanine, and taurine, were the most enriched metabolites in DCM hearts, while 4-aminobenzoate (alias PABA or vitamin B10), 6-phosphogluconate, and creatine were the less enriched ones. Pathway enrichment and pathway topology analyses showed that alterations in glycolysis, pyruvate metabolism, CoA synthesis, and citrate cycle are consistent with the metabolome of DCM (Table S4). Branched chain amino acid (valine, leucine, and isoleucine) metabolism is also possibly altered in DCM. Since BCAAs are potent activators of mTOR and may disrupt cardiac glucose metabolism [28], we analyzed the expression of PP2Cm, a protein phosphatase regulating the function of the rate-limiting enzyme of BCAA catabolism. PP2Cm levels were significantly reduced in DCM samples (Figure 4C). Conversely, the levels of miR-22, an inhibitor of PP2Cm expression [29] that is upregulated by hypertrophic stimuli [30] and suppresses cardiomyocyte autophagy [31], was highly enriched in DCM samples. Importantly, miR-22 expression was significantly correlated with the nuclear expression of the redox sensitive transcriptional regulator APE/Ref (*p* = 0.46, *p* = 0.009).

Altogether, these results suggest that miR-22 overexpression is coupled with complex alterations of cardiac metabolism, leading to the accumulation of BCAA, and is possibly linked to alteration of glucose utilization and suppression of autophagy.

### 3.5. Alterations of the Autophagic Process and Mitochondrial Function are Coupled with Accelerated Senescence of Cardiac Pericytes in DCM

**Figure 5:**
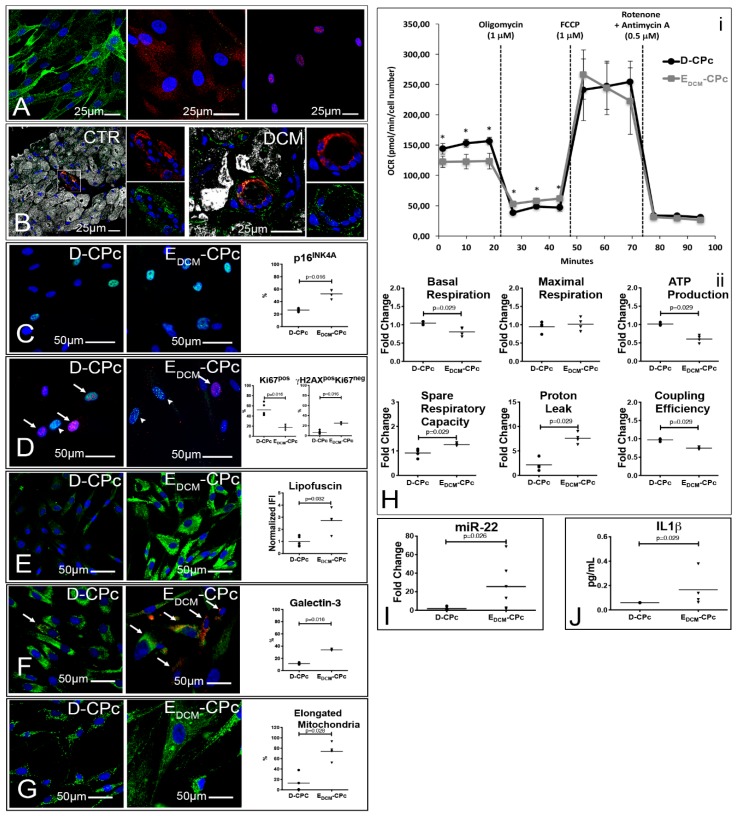
Cardiac pericytes isolated from diseased hearts are senescent and accumulate dysfunctional mitochondria. (**A**,**B**) In vitro and in vivo characterizations of CPc. Confocal images of cultured CPc (*n* = 5 D-CPc and *n* = 5 E_DCM_-CPc) stained for NG2 (green, left panel), PDGFRα and PDGFRβ (green and red, central panel), and Tbx18 (red, right panel; (**A**)). Confocal images showing that mostly PDGFRβ^+^ cells (green) are localized in and around arterioles and capillaries and coexpress smooth muscle actin (red). ASA is shown in white (**B**). (**C**–**G**) Senescence and ALP dysfunction of E_DCM_-CPc. Epifluorescence images of CPc isolated from normal (D-CPc; *n* = 5) and pathologic hearts (E_DCM_-CPc; *n* = 4) show positivity to p16^INK4A^ (green, C), γH2A.X (green) and Ki67 (red, **D**), lipofuscins (green, €), Galectin 3 (red) and LAMP2 (green, (**F**)), and mitochondria (green, (**G**)). Nuclei are labeled by DAPI in blue. Dot plots in C–G indicate the results of the quantitative analysis; Mann–Whitney tests were employed. (H) Profiles of mitochondria bioenergetics measurements. Plots in (i) display average (±S.D.) OCR of 4 D- and 4 E_DCM_-CPc, measured at baseline and after the addition of the stressors oligomycin, FCCP, rotenone, and antimycin A. Time and type of stressor administration are indicated by dashed lines. Parameters quantified from the experiment were normalized employing the respective average value of D-CPc and are shown in dot plots in (ii); Mann–Whitney tests were employed. (**I**,**J**) Real-time PCR and ELISA analyses of CPc. Results of RT-PCR analysis of miR22 expression in 6 D- and 6 E_DCM_-CPc (**I**). IL1β concentration in CPc culture supernatant (**J**); Mann–Whitney tests were employed.

Last, since many of the crucial alterations observed in myocytes could also be observed in interstitial stromal cells (Figure S2), we isolated and cultured these cells in order to support histological findings with functional data. Cultured stromal cells were characterized as being NG2^+^PDGFR**β**^+^PDGFR**α**^−/low^Tbx18^+^ cardiac pericytes and were obtained both from atria of explanted DCM hearts (E_DCM_-CPc) and from normal, donor hearts (D-CPc); Figure 5A. In vivo, PDGFR**β**^+^ cells display both a mural/perivascular and interstitial location (Figure 5B).

With respect to D-CPc, E_DCM_-CPc were more senescent (either p16^+^ or **γ**H2AX^+^Ki67^−^ Figure 5C–D) and less proliferative (Ki67^+^, Figure 5D). Importantly, E_DCM_-CPc accumulated lipofuscins in the cytoplasm, showed evidence of LMP (Figure 5E–F), and possessed very elongated and branched mitochondria (Figure 5G). These morphological differences were paralleled by functional differences in mitochondrial respiration; E_DCM_-CPc cell lines presented a significantly lower basal OCR, a significantly reduced ATP production and coupling efficiency, and a significantly increased proton leak (Figure 5H). Furthermore, increased miR22 expression (Figure 5I) and increased IL1**β** secretion (Figure 5J) characterized E_DCM_-CPC.

**Figure 6:**
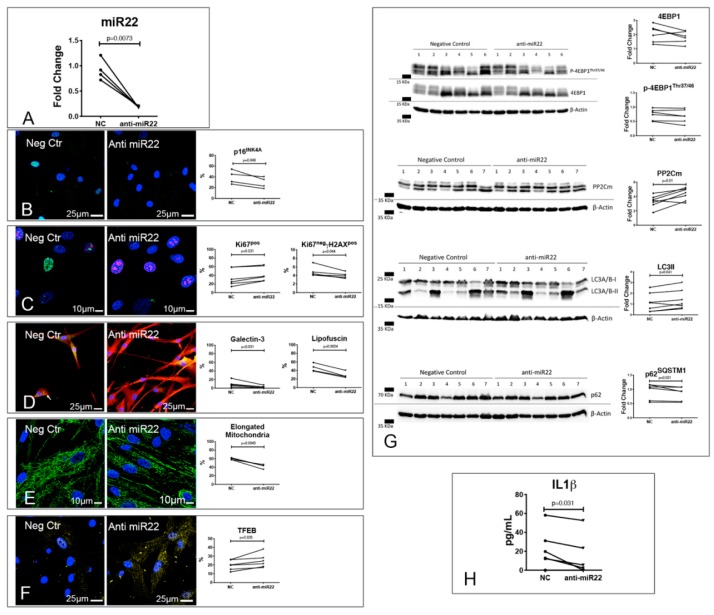
Inhibition of miR-22 reverses E_DCM_-CPc senescence. Results of RT-PCR analysis of E_DCM_-CPc treated with either anti-miR22 or a negative control (NC; (**A**)). (**B**–**F**) Reversal of E_DCM_-CPc senescence and ALP dysfunction by miR-22 inhibition. Confocal images of E_DCM_-CPc treated with either anti-miR22 or NC showing positivity to p16INK4A (green, (**B**)), γH2A.X (green) and Ki67 (red, (**C**)), lipofuscins (green) and Galectin 3 (red, **D**), mitochondria (green, (**E)**), and TFEB (yellow, (**F**)). Nuclei are labeled by DAPI in blue. (**G**) Effects of miR-22 inhibition on mTOR targets, PP2Cm, and autophagy. Representative WB of whole protein extracts of 6–7 E_DCM_-CPc treated with either anti-miR22 or NC (**G**). Blotted proteins were incubated with antibodies against 4EBP1, p-4EBP1^Thr37/46^, PP2Cm, LC3A/B or p62^SQSTM1^. Results of the quantitative analyses are reported in the right panels. (H) Effects of miR-22 inhibition on IL1β secretion. ELISA assay was used for the detection of IL1β released in the culture supernatant of 7 E_DCM_-CPc treated with either anti-miR22 or NC (**H**). Dot plots indicate the results of quantitative analyses; paired *t* tests (p16^INK4A^, Ki67, lipofuscin, mitochondria, TFEB, 4EBP1, p-4EBP1^Thr37/46^, PP2Cm, and LC3A/B) or Wilcoxon tests (Ki67^−^γH2A.X^+^, Galectin 3, p62^SQSTM1^, and IL1β) were employed, as appropriate.

To directly assess the impact of miR-22 in CPc biology, we first inhibited it in patient-derived CPc, employing an anti-miR for 3 d (Figure 6A). By down-regulating miR22, we significantly reduced the frequency of senescent E_DCM_-CPc (Figure 6B,C) and increased the frequency of proliferating E_DCM_-CPc (Figure 6C). These changes were associated with a significant reduction of the frequency of E_DCM_-CPc showing evidence of LMP, lipofuscin accumulation (Figure 6D), and elongated mitochondria (Figure 6E). Moreover, anti-miR22 treatment increased the frequency of E_DCM_-CPc showing nuclear localization of TFEB (Figure 6F). These functional modifications of E_DCM_-CPc were associated with a trend to reduced 4EBP-1 levels (*p* = 0.10), coupled with significantly increased levels of both PP2Cm and lipidated LC3, and decreased levels of p62^SQSTM1^ (Figure 6G), supporting the relevant role played by miR-22 in autophagy inhibition. Importantly, by inhibiting miR22 in E_DCM_-CPc, we were able to reduce the levels of IL1β released in their culture supernatant (Figure 6H).

Intriguingly, when we exposed CPc derived from normal hearts to a short (3 d) treatment with a miR-22 mimic, we were able to recapitulate only marginally the alterations observed in CPc isolated from failing DCM hearts. Specifically, although we did not observe a clear effect on proliferation and senescence, we documented a significant reduction in cell density, which is suggestive of ongoing cell death, associated with an alteration of mitochondrial morphology, TFEB nuclear translocation, and lipofuscin accumulation (Figure S3).

Altogether, these results point to the central role of miR-22 overexpression in dictating E_DCM_-CPC cell senescence, loss of proteostasis, and mitochondrial dysfunction.

## 4. Discussion

Since IL1β plasma levels can predict long-term prognosis of ambulatory patients affected by DCM [16], understanding the mechanisms leading to inflammation is critical to dissect the clinical variability of DCM patients. To be secreted, IL1β needs to be transcribed (mostly in a NFκB-dependent fashion) [32]. Consistently, we observed that NFκB, miR-146a, and miR-146b are more expressed in DCM hearts. Pro-IL1β is subsequently processed by active caspase1, which is activated by the inflammasome and is required for IL1β and IL18 secretion [33]. Inflammasomes are activated by sensor proteins (e.g., NLRP3), which are triggered by DAMPs (e.g., mitochondrial ROS and DNA or cathepsin release following LMP) leading to sterile inflammation [33].

To verify how DAMPs could accumulate in DCM, we focused on proteostasis, a network of molecular (e.g., chaperones, and the UPS), organelle (i.e., the ALP), intercellular (i.e., exosomes and tunneling nanotubes), and (possibly) interorgan pathways preventing the accumulation of unwanted proteomic changes [34]. Consistently with literature data [11], we observed that amyloid-like substances and poly-ubiquitinated proteins accumulated in DCM hearts. These alterations were coupled with ALP impairment and aggresome accumulation [13], in analogy with results obtained by experimentally inhibited autophagy [35]. Mitophagy and ALP are crucial for mitochondrial quality control [36]. Consistently, we showed that dysfunctional, Parkin-labeled mitochondria [36] and 4HNE (a product of cardiolipin peroxidation), which are potent NLRP3 triggers [33], accumulated in DCM.

Concerning ALP impairment, we observed both reduced Beclin1 expression and nuclear translocation of TFEB, coupled with evidence of LMP, in DCM hearts. Notably, the presence of an active mTOR Complex1 (mTORC1) in DCM samples could account for many of the above reported alterations, since it suppresses lysosomal biogenesis, promoting the exclusion of TFEB from the nucleus [23], and inhibits autophagy, promoting both ULK1 phosphorylation [37] and inhibition of TFEB-dependent transcription of autophagy genes [38,39]. Since mTOR is sensitive to the availability of nutrients [37], and DCM that evolves to heart failure is characterized by a pathologic alteration of cardiac metabolism [27], we analyzed the metabolome of normal and DCM hearts. In explanted hearts, we observed both a shift from oxidative to glycolytic metabolism and the accumulation of the BCAAs leucine and valine, two of the best characterized activators of mTORC1 [37], which can alter glucose metabolism too [28]. This was coupled with a reduced expression of PP2Cm, a mitochondrial targeted phosphatase that inhibits the enzymatic complex catalyzing the rate-limiting step of BCAA catabolism [40]. Consistently, patients affected by DCM display alterations of BCAA catabolism, while *PP2Cm* gene deletion exerts a negative impact on mouse heart function [41]. Importantly, miR22, a strong inhibitor of the autophagic process [31] whose ability to downregulate PP2Cm levels has been validated [29], is upregulated in DCM samples.

Finally, we analyzed cardiac pericytes, since they are the second most common cell type in the heart [42], whose involvement in cardiac pathology is emerging. Indeed, pericytes are important regulators of the cardiac microvascular blood flow [43], whose dysfunction also characterizes DCM [44], plays a role in fibrosis [45], and are possible sentinels of the innate immunity [46]. We observed that E_DCM_-CPc are characterized by increased senescence rates, lysosomal dysfunction, and lipofuscin accumulation, coupled with mitochondrial dysfunction, miR22 upregulation, and IL1β secretion. To directly prove the involvement of miR22 in modulating CPc biology, we first silenced its expression in E_DCM_-CPc. Consistently with our hypothesis, miR22 downregulation was associated with a significant upregulation of PP2Cm expression, confirming literature data [29] in this specific cell type. We also observed a trend to attenuate mTOR signaling and a significant reprise of autophagy, as shown by the increased nuclear localization of TFEB, the increase in LC3-II levels, and decrease in p62^SQSTM1^ levels. These modifications were coupled with a decrease in the frequency of cells showing evidence of LMP and accumulation of elongated mitochondria and lipofuscins. Most importantly, miR22 inhibition reduced the secretion of IL1β in E_DCM_-CPc culture supernatant. Conversely, an acute overexpression of miR22 in healthy CPc mainly resulted in an alteration of mitochondrial dynamics. We could speculate that, given the important role of PP2Cm in regulating the mitochondrial permeability transition pore opening and cell survival [47], those healthy cells that survived miR22 overexpression mainly responded to this acute stimulus by increasing mitochondrial fragmentation and, as a response, promoting TFEB nuclear localization, which is consistent with literature data [48]. The accumulation of lipofuscins in miR22-overexpressing cells may be considered additional indirect evidence of altered mitochondrial dynamics, as in [49].

## 5. Conclusions

**Figure 7:**
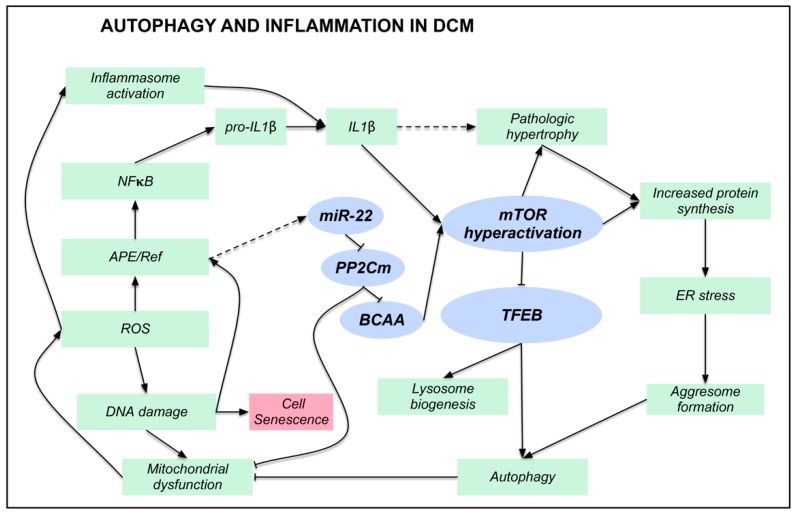
Autophagy and inflammation in DCM. Illustration summarizing the main findings described in human DCM samples. Blue ellipses indicate the key actors of this process. Dashed arrows indicate correlative or literature findings. Arrows with blunt ends indicate inhibition, while pointed arrows indicate activation.

A complex series of events, linking alterations of proteostasis with inflammation (Figure 7), characterizes DCM. miR22 overexpression, PP2Cm downregulation, BCAA accumulation, mTOR hyperactivation, and ALP suppression are key actors of this process. The significant correlation between TFEB expression and both disease duration and left ventricular ejection fraction further support the idea that interfering with this process could modify the clinical history of DCM.

## Figures and Tables

**Table 1:** Clinical, demographic, and anatomical parameters of the patients whose hearts were included in the histology study.

	Cardiomyopathy (*n* = 50)	Controls (*n* = 18)	Normal Values	*p*
Age (Yr)	51.6 ± 13.9	40.7 ± 12.4	-	0.0068
Sex (M/F)	40/10	8/10	-	0.0072
Duration of disease (Years)	8.36 ± 6.54	-	-	
NYHA class (%)				
II	9	-	-	-
III	67	-	-	-
IV	24	-	-	-
Echocardiography §				
Left ventricular diameter (mm)				
Systolic	62.7 ± 12.1	-	21.6–34.8	-
Diastolic	74.0 ± 11.3	-	37.8–52.2	-
Left ventricular Volumes (mL)				
End Diastolic	197 ± 75	-	46–106	-
End Systolic	149 ± 60	-	14–42	-
LV Ejection Fraction (%)	23 ± 7	-	54–74	-
Hemodynamics ‡				
Pulmonary Artery Pressure (mmHg)				
Systolic	41.92 ± 13.59	-	15–25	-
Diastolic	22.15 ± 10.31	-	8–12	-
Mean	29.45 ± 11.18	-	10–20	-
PCWP (mmHg)	21.56 ± 10.42	-	6–12	-
CI (L⋅min^−1^⋅m^−2^)	2.37 ± 0.73	-	2.5–4.0	-
Gross Anatomy †				
Heart Weight (g)	494 ± 161	334 ± 109	196–516	0.0011
Transverse diameter (mm)	129 ± 16	100 ± 20	-	<0.0001
Inner longitudinal diameter (mm)	94 ± 16	73 ± 3	-	<0.0001
Wall thickness (mm)				
LV	9.5 ± 2.1	13 ± 1.2	-	0.0073
RV	4.1 ± 1.9	3.3 ± 1.5	-	n.s.
Septum	11.5 ± 2.4	12.0 ± 2.0	-	n.s.
Comorbidities				
BMI>30 (%)	7.5			
Arrhythmia (%)	75.6			
Mitral Insufficiency (%)	82.6			
Chronic Kidney Failure (%)	4.5			
BPCO (%)	6.8			
Smoke	4.5			
Hypothyroidism	9.1			
Hyperthyroidism	2.3			
Hypertension (%)	2.3			
Dyslipidemia (%)	20.5			
Diabetes (%)	11.4			
Pharmacological therapy				
ACE-I/ARB (%)	82.5			
β-Blockers (%)	58.5			
Digitalis (%)	80.5			
Dobutamine (%)	30			
Amiodarone (%)	56.1			
Antialdosteronic (%)	67.5			
K Sparing diuretics (%)	12.5			
Loop diuretics (%)	97.5			
Insulin / Antidiabetics (%)	7.5			
Statins (%)	0			
Oral Anticoagulants (%)	57.5			
Tiroxin (%)	9.8			

**Legend:** NYHA- New York Heart Association Functional Classification; Normal value as in: (§) Lang, R.M.; Badano, L.P.; Mor-Avi, V.; Afilalo, J.; Armstrong, A.; Ernande, L.; Flachskampf, F.A.; Foster, E.; Goldstein, S.A.; Kuznetsova, T.; et al. Recommendations for cardiac chamber quantification by echocardiography in adults: An update from the American Society of Echocardiography and the European Association of Cardiovascular Imaging. *Eur. Heart J. Cardiovasc. Imaging*. **2015**, *16*, 233–270. (‡) Bangalore, S.; Bhatt, D.L. Images in cardiovascular medicine. Right heart catheterization, coronary angiography, and percutaneous coronary intervention. *Circulation* **2011**, *124*, e428–433. (†) Sheppard, M. *Practical Cardiovascular Pathology*, 2nd ed.; Taylor & Francis: London, UK, 2011.

**Table 2:** Clinical, demographic, and anatomical parameters of the patients whose hearts were included in the metabolomic study.

	Cardiomyopathy (*n* = 12)	Controls (*n* = 12)	Normal Values	*p*
Age (Yr)	57.9 ± 6.1	46.9 ± 15.9	-	0.04
Sex (M/F)	11/1	6/6	-	n.s.
Duration of disease (Years)	23.9 ± 32.1	-	-	
NYHA class (%)				
III	75	-	-	-
IV	25	-	-	-
Echocardiography §				
Left ventricular diameter (mm)				
Systolic	61.6 ± 11.9	27.1 ± 5.0	21.6–34.8	<0.0001
Diastolic	71.0 ± 7.9	45.4 ± 4.5	37.8–52.2	<0.0001
Left ventricular Volumes (mL)				
End Diastolic	220.2 ± 112.4	92.0 ± 50.4	46–106	0.047
End Systolic	170.6 ± 98.9	40.7 ± 23.1	14–42	0.012
LV Ejection Fraction (%)	22.8 ± 8.4	63.4 ± 5.8	54–74	<0.0001
Hemodynamics ‡				
Pulmonary Artery Pressure (mmHg)				
Systolic	45.9 ± 15.2	-	15–25	
Diastolic	20.7 ± 11.6	-	8–12	
Mean	30.0 ± 13.8	-	10–20	
PCWP (mmHg)	18.5 ± 11.4	-	6–12	
CI (L⋅min^−1^⋅m^−2^)	1.9 ± 0.36	-	2.5–4.0	
Gross Anatomy †				
Heart Weight (g)	555.4 ± 194.5	-	196–516	
Transverse diameter (mm)	125 ± 12	-	-	
Inner longitudinal diameter (mm)	93 ± 13	-	-	
Wall thickness (mm)				
LV	11.6±1.8	-	-	
RV	7.2±3.5	-	-	
Septum	13.9±3.5	-	-	
Comorbidities				
BMI>30 (%)	8	-		
Arrhythmia (%)	90	-		
Mitral Insufficiency (%)	88	-		
Chronic Kidney Failure	40	-		
BPCO (%)	10	**-**		
Smoke	0	**-**		
Hypothyroidism	10	**-**		
Hyperthyroidism	20	**-**		
Hypertension (%)	30	-		
Dyslipidemia (%)	40	-		
Diabetes (%)	20	-		
Pharmacological therapy				
ACE-I/ARB (%)	66.7	-		
β-Blockers (%)	80	-		
Digitalis (%)	60	-		
Dobutamine (%)	10	-		
Amiodarone (%)	30	**-**		
Antialdosteronic (%)	40	**-**		
K Sparing diuretics (%)	50	**-**		
Loop diuretics (%)	80	**-**		
Insulin / Antidiabetics (%)	10	-		
Statins (%)	30	-		
Oral Anticoagulants (%)	70	-		
Tiroxin (%)	20	-		

Normal value as in: (§) Lang, R.M.; Badano, L.P.; Mor-Avi, V.; Afilalo, J.; Armstrong, A.; Ernande, L.; Flachskampf, F.A.; Foster, E.; Goldstein, S.A.; Kuznetsova, T.; et al. Recommendations for cardiac chamber quantification by echocardiography in adults: An update from the American Society of Echocardiography and the European Association of Cardiovascular Imaging. *Eur. Heart J. Cardiovasc. Imaging*. **2015**, *16*, 233–270. (‡) Bangalore, S.; Bhatt, D.L. Images in cardiovascular medicine. Right heart catheterization, coronary angiography, and percutaneous coronary intervention. *Circulation* **2011**, *124*, e428–433. (†) Sheppard, M. *Practical Cardiovascular Pathology*, 2nd ed.; Taylor & Francis: London, UK, 2011.

## Supplementary Materials

The following are available online at https://www.mdpi.com/2077-0383/8/10/1519/s1, Figure S1: WB of whole protein extracts of 7 DCM heart and 7 autoptic controls, Figure S2: Quantitation of immunofluorescence stainings on α-sarcomeric actin negative cells, Figure S3: Effects of miR22 overexpression on D-CPc, Table S1: Clinical, demographic, and anatomical parameters of the patients and autoptic controls whose hearts were included in the supplementary Figure S1, Table S2: Antibodies employed for histology, cellular, and molecular studies, Table S3: Univariate analysis of 22 metabolites unambiguously identified by 1D NMR spectroscopy, Table S4: Pathway analysis of metabolites unambiguously identified by 1D NMR.
