# Supplementary material for: Autophagy and Inflammasome Activation in Dilated Cardiomyopathy
Source: J Clin Med. 2019 Sep 21;8(10):1519. doi: 10.3390/jcm8101519 (PMC6832472; doi:10.3390/jcm8101519)
Supplement: Supplementary file 1 [file jcm-08-01519-s001.zip › Supplementary Material/Supplementary Table 3.docx]

**Supplementary Table 3: Univariate analysis of 22 metabolites unambiguously identified by 1D NMR spectroscopy.**

| Metabolite | Bucket (ppm) | Assignment | Mean Ctrl | SD Ctrl | Mean DCM | SD DCM | *p* | DCM/Ctrl |
| --- | --- | --- | --- | --- | --- | --- | --- | --- |
| 4-Aminobenzoic acid | 7.70 .. 7.72 | D 2xCH | 0.256 | 0.522 | -0.256 | 0.051 | 0.00597* | Down |
| 6-Phosphogluconic acid | 4.16 .. 4.18 | D CH2 | 0.991 | 1.838 | -0.991 | 0.201 | 0.0033* | Down |
| Acetic acid | 1.90 .. 1.92 | S CH3 | -0.595 | 2.518 | 0.595 | 6.325 | 0.67066 | Up |
| Alanine | 1.46 .. 1.47 | D CH3 | -0.671 | 2.804 | 0.671 | 1.817 | 0.17823 | Up |
| AMP | 6.11 .. 6.13 | D CH | -0.177 | 0.569 | 0.177 | 0.602 | 0.19781 | Up |
| Aspartic acid | 2.81 .. 2.84 | DD CH2 beta | 0.251 | 0.947 | -0.251 | 0.748 | 0.16408 | Down |
| Creatine | 3.90 .. 3.93 | S CH2 | 1.712 | 3.49 | -1.712 | 1.299 | 0.00661* | Down |
| Fumaric acid | 6.50 .. 6.52 | S 2xCH | -0.168 | 0.202 | 0.168 | 0.401 | 0.03872* | Up |
| Glucose | 4.63 .. 4.65 | D CH anomeric beta | -0.13 | 0.406 | 0.13 | 0.469 | 0.16041 | Up |
| Glutamic acid | 2.35 .. 2.37 | M CH2 gamma | -0.163 | 1.413 | 0.163 | 1.583 | 0.59922 | Up |
| Glutamine | 2.40 .. 2.42 | M CH2 gamma | -0.243 | 0.75 | 0.243 | 0.666 | 0.10734 | Up |
| Glycine | 3.56 .. 3.58 | S CH2 | 0.395 | 1.876 | -0.395 | 1.446 | 0.25979 | Down |
| Isoleucine | 1.00 .. 1.02 | D CH3 | -0.125 | 0.381 | 0.125 | 0.345 | 0.10563 | Up |
| Lactic acid | 4.11 .. 4.14 | Q CH | -0.826 | 1.61 | 0.826 | 0.72 | 0.00535* | Up |
| Leucine | 0.93 .. 0.96 | T 2xCH3 | -0.432 | 0.859 | 0.432 | 0.777 | 0.01687* | Up |
| Myoinositol | 4.04 .. 4.06 | T CH | 0.963 | 3.386 | -0.963 | 1.788 | 0.12769 | Down |
| NAD+ | 8.15 .. 8.17 | S CH | -0.127 | 0.398 | 0.127 | 0.543 | 0.20621 | Up |
| Nicotinic acid | 8.92 .. 8.94 | S CH | 0.076 | 0.315 | -0.076 | 0.234 | 0.19361 | Down |
| Phenylalanine | 7.30 .. 7.32 | D CH | -0.206 | 0.235 | 0.206 | 0.354 | 0.00281* | Up |
| Pyruvic acid | 2.38 .. 2.40 | S CH3 | -1.403 | 0.825 | 1.403 | 1.702 | 0.0001* | Up |
| Taurine | 3.39 .. 3.40 | T CH2 | -1.043 | 1.817 | 1.043 | 2.593 | 0.03243* | Up |
| Valine | 1.03 .. 1.05 | D CH3 | -0.33 | 0.338 | 0.33 | 0.531 | 0.00149* | Up |
