# Supplementary material for: Autophagy and Inflammasome Activation in Dilated Cardiomyopathy
Source: J Clin Med. 2019 Sep 21;8(10):1519. doi: 10.3390/jcm8101519 (PMC6832472; doi:10.3390/jcm8101519)
Supplement: Supplementary file 1 [file jcm-08-01519-s001.zip › Supplementary Material/Supplementary Table 4.docx]

**Supplementary Table 4: Pathway analysis of metabolites unambiguously identified by 1D NMR.**

| Pathway | Total Cmpd | Hits | Raw p | -LOG(p) | Impact |
| --- | --- | --- | --- | --- | --- |
| Pentose phosphate pathway | 32 | 3 | 0.00025913 | 8.2582 | 0.13556 |
| Phenylalanine metabolism | 45 | 3 | 0.0036234 | 5.6203 | 0.11906 |
| Citrate cycle (TCA cycle) | 20 | 2 | 0.0036602 | 5.6102 | 0.10708 |
| Butanoate metabolism | 40 | 2 | 0.0036602 | 5.6102 | 0.10287 |
| Tyrosine metabolism | 76 | 2 | 0.0036602 | 5.6102 | 0 |
| Pantothenate and CoA biosynthesis | 27 | 2 | 0.0037024 | 5.5988 | 0 |
| Nicotinate and nicotinamide metabolism | 44 | 4 | 0.0041936 | 5.4742 | 0.10715 |
| Valine, leucine and isoleucine biosynthesis | 27 | 3 | 0.0058458 | 5.142 | 0.04823 |
| Alanine, aspartate and glutamate metabolism | 24 | 4 | 0.0072365 | 4.9286 | 0.23267 |
| Ascorbate and aldarate metabolism | 45 | 2 | 0.0078178 | 4.8514 | 0.01617 |
| Arginine and proline metabolism | 77 | 4 | 0.018835 | 3.972 | 0.03163 |
| Glycine, serine and threonine metabolism | 48 | 3 | 0.022105 | 3.8119 | 0.18845 |
| Pyruvate metabolism | 32 | 3 | 0.058325 | 2.8417 | 0.41957 |
| Glycolysis or Gluconeogenesis | 31 | 4 | 0.058753 | 2.8344 | 0.09576 |
| Taurine and hypotaurine metabolism | 20 | 3 | 0.059253 | 2.8259 | 0.35252 |
| Propanoate metabolism | 35 | 2 | 0.061985 | 2.7809 | 0 |
| Valine, leucine and isoleucine degradation | 40 | 2 | 0.080738 | 2.5165 | 0.02232 |
| Primary bile acid biosynthesis | 47 | 2 | 0.10642 | 2.2404 | 0.01644 |
| Nitrogen metabolism | 39 | 5 | 0.11065 | 2.2014 | 0 |
| Aminoacyl-tRNA biosynthesis | 75 | 5 | 0.20558 | 1.5819 | 0 |
| Galactose metabolism | 41 | 2 | 0.28099 | 1.2694 | 0.00276 |
| Cyanoamino acid metabolism | 16 | 2 | 0.37427 | 0.98278 | 0 |
| Purine metabolism | 92 | 3 | 0.52944 | 0.63594 | 0.08152 |
