# Supplementary material for: Autophagy and Inflammasome Activation in Dilated Cardiomyopathy
Source: J Clin Med. 2019 Sep 21;8(10):1519. doi: 10.3390/jcm8101519 (PMC6832472; doi:10.3390/jcm8101519)
Supplement: Supplementary file 1 [file jcm-08-01519-s001.zip › Supplementary Material/Supplementary table 2.pdf]

Supplementary Table2: Antibodies and experimental conditions employed for histological, cellular and Western Blotting studies.

Histology

Antigen	Company	Dilution	Antigen retrieval	Incubation time	Incubation temperature	Secondary antibody
Poly-Ubiquitinated proteins	Enzo Lifescience	1:300	pH6	1h	RT	IHC (EnVision)
Vimentin	Abcam	1:100	pH6	2h	37°C	A488 anti Rabbit
p62SQSTM1	MBL	1:500	pH6	1h	RT	IHC (EnVision)
LAMP2	Abcam	1:20	pH6	1h	37°C	A488 anti Mouse
Galectin3	R&D	1:200	pH6	O/N	4°C	A555 anti Goat
TFEB	Abcam	1:200	pH9	O/N	4°C	A555 anti Rabbit
Parkin	Biorbyt	1:100	pH9	1h	RT	IHC (EnVision)
4-hydroxynonenal	Bioss	1:500	pH6	1h	RT	IHC (EnVision)
53BP1	Abcam	1:50	pH6	O/N	4°C	A488 anti Rabbit
APE/Ref-1	Kind gift of Prof. G. Tell	1:100	pH6	1h	RT	IHC (EnVision)
p65 RelA	Abcam	1:500	pH6	O/N	4°C	IHC (EnVision)
IL1ß	Abcam	1:50	pH9	O/N	4°C	IHC (EnVision)
NLRP3	Cell Signaling	1:500	pH9	O/N	4°C	A488 anti Rabbit
Caspase1	ThermoFisher	1:600	pH9	1h	37°C	A555 anti Mouse
PDGFRβ	ThermoFisher	1:50	pH6	O/N	4°C	A555 anti Rabbit
Alpha-Smooth Muscle Actin	Dako	1:100	pH6	1h	37°C	A488 anti Mouse
Alpha-Sarcomeric Actin	Sigma	1:400	pH6 or pH9	1h	37°C	Cy5 anti Mouse IgM

Cytology

Antigen	Company	Dilution	Antigen retrieval	Incubation time	Incubation temperature	Secondary antibody
NG2	ThermoFisher	1:100	Triton X, 1:100	O/N	4°C	A488 anti Mouse
PDGFRα	ThermoFisher	1:100	Triton X, 1:100	2h	37°C	A488 anti Mouse
PDGFRβ	ThermoFisher	1:100	Triton X, 1:100	O/N	4°C	A555 anti Rabbit
Tbx18	ThermoFisher	1:100	Triton X, 1:100	O/N	4°C	A555 anti Rabbit
p16INK4A	CIN-TEK	1:2	Triton X, 1:100	O/N	4°C	A488 anti Mouse
γH2A.X	Millipore	1:500	Triton X, 1:100	2h	37°C	A555 anti Mouse
Ki67	Leica-Novocastra	1:1000	Triton X, 1:100	O/N	4°C	A488 anti Rabbit
Galectin3	R&D	1:1000	Triton X, 1:100	O/N	4°C	A647 anti Goat
LAMP2	Abcam	1:100	Triton X, 1:100	2h	37°C	A555 anti Mouse
Parkin	Biorbyt	1:50	Triton X, 1:100	O/N	4°C	A594 anti Rabbit
Mitochondria	NeoMarkers	1:10	Triton X, 1:100	2h	37°C	A488 anti Mouse
TFEB	Cell Signaling	1:600	Triton X, 1:100	O/N	4°C	A555 anti Rabbit

Western Blotting

Antigen	Company	Host	Clonality	Dilution	Incubation time	Incubation temperature
PPM1K	ABCAM	RABBIT	POLYCLONAL	1:1000	OVERNIGHT	+4 °C
AKT	CELL SIGNALING	RABBIT	MONOCLONAL	1:1000	OVERNIGHT	+4 °C
Phospho-AKT (Ser473)	CELL SIGNALING	RABBIT	MONOCLONAL	1:1000	OVERNIGHT	+4 °C
BECLIN 1	CELL SIGNALING	RABBIT	MONOCLONAL	1:1000	OVERNIGHT	+4 °C
ATG 5	CELL SIGNALING	RABBIT	POLYCLONAL	1:1000	OVERNIGHT	+4 °C
ATG7	CELL SIGNALING	RABBIT	POLYCLONAL	1:1000	OVERNIGHT	+4 °C
mTOR	CELL SIGNALING	RABBIT	POLYCLONAL	1:1000	OVERNIGHT	+4 °C
Phospho-mTOR (Ser2481)	CELL SIGNALING	RABBIT	POLYCLONAL	1:1000	OVERNIGHT	+4 °C
Phospho-mTOR (Ser2448)	CELL SIGNALING	RABBIT	POLYCLONAL	1:1000	OVERNIGHT	+4 °C
Actin	SIGMA	RABBIT	POLYCLONAL	1:2000	1h	RT
GAPDH	CELL SIGNALING	RABBIT	MONOCLONAL	1:2000	OVERNIGHT	+4 °C
AMPKα	CELL SIGNALING	RABBIT	MONOCLONAL	1:1000	OVERNIGHT	+4 °C
Phospho-AMPKα (Thr172)	CELL SIGNALING	RABBIT	MONOCLONAL	1:1000	OVERNIGHT	+4 °C
p70 S6 Kinase	CELL SIGNALING	RABBIT	POLYCLONAL	1:1000	OVERNIGHT	+4 °C
Phospho-p70 S6 Kinase (Thr389)	CELL SIGNALING	RABBIT	POLYCLONAL	1:500	OVERNIGHT	+4 °C
4E-BP1	CELL SIGNALING	RABBIT	MONOCLONAL	1:1000	OVERNIGHT	+4 °C
Phospho-4E-BP1 (Thr37/46)	CELL SIGNALING	RABBIT	MONOCLONAL	1:1000	OVERNIGHT	+4 °C
Parkin	CELL SIGNALING	MOUSE	MONOCLONAL	1:1000	OVERNIGHT	+4 °C
LC3A/B	CELL SIGNALING	RABBIT	MONOCLONAL	1:1000	OVERNIGHT	+4 °C
SQSTM1/p62	CELL SIGNALING	MOUSE	MONOCLONAL	1:1000	OVERNIGHT	+4 °C

Legend:

pH6	Flex-Target Retrieval Solution (Agilent) - Low pH, 40' at 98°C
pH9	Flex-Target Retrieval Solution (Agilent) - High pH, 40' at 98°C
A488	Alexa 488 labeled donkey Antibody
A555	Alexa 555 labeled donkey Antibody
A647	Alexa 647 labeled donkey Antibody
Cy5	Cy5 labeled donkey Antibody
