# Supplementary material for: Autophagy and Inflammasome Activation in Dilated Cardiomyopathy
Source: J Clin Med. 2019 Sep 21;8(10):1519. doi: 10.3390/jcm8101519 (PMC6832472; doi:10.3390/jcm8101519)
Supplement: Supplementary file 1 [file jcm-08-01519-s001.zip › Supplementary Material/Suppl Tab1.pdf]

Supplementary Table 1. Clinical, demographic and anatomical parameters of the patients whose hearts were included in the metabolomic study.

	Cardiomyopathy (n=7)	Controls (n=7)	Normal Values	p
**Age (Yr)**	49.0±13.0	81.6±11.2	-	<0.001
**Sex (M/F)**	5/2	2/5	-	n.s.
**Duration of disease (Years)**	11.1±6.7	-	-	
**NYHA class (%)**				
**III**	67	-	-	-
**IV**	33	-	-	-
		**Echocardiography §**		
**Left ventricular diameter (mm)**				
**Systolic**	69±11	-	21.6-34.8	-
**Diastolic**	73±10	-	37.8-52.2	-
**LV Ejection Fraction (%)**	21.1±12.3	-	54-74	-
		**Gross Anatomy †**		
**Heart Weight (g)**	438.9±176.9	-	196-516	
**Transverse diameter (mm)**	117±17	-	-	
**Inner longitudinal diameter (mm)**	94±18	-	-	
**Wall thickness (mm)**				
**LV**	9±1.3	-	-	
**RV**	7.0 ±2.7	-	-	
**Septum**	13.0±1.9	-	-	
		**Pharmacological therapy**		
**ACE-I/ARB (%)**	100	-		
**β-Blockers (%)**	83	-		
**Digitalis (%)**	20	-		
**Dobutamine (%)**	33	-		
**Amiodarone (%)**	40	-		
**Antialdosteronic (%)**	40	-		
**K Sparing diuretics (%)**	60	-		
**Loop diuretics (%)**	80	-		
**Insulin / Antidiabetics (%)**	20	-		
**Statins (%)**	0	-		
**Oral Anticoagulants (%)**	20	-		
**Tiroxin (%)**	20	-		

Normal value as in: (§) Lang RM, Badano LP, Mor-Avi V, Afilalo J, Armstrong A, Ernande L, Flachskampf FA, Foster E, Goldstein SA, Kuznetsova T, Lancellotti P, Muraru D, Picard MH, Rietzschel ER, Rudski L, Spencer KT, Tsang W and Voigt JU. Recommendations for cardiac chamber quantification by echocardiography in adults: an update from the American Society of Echocardiography and the European Association of Cardiovascular Imaging. *Eur Heart J Cardiovasc Imaging*. 2015;16:233-70; (‡) Bangalore S and Bhatt DL. Images in cardiovascular medicine. Right heart catheterization, coronary angiography, and percutaneous coronary intervention. *Circulation*. 2011;124:e428-33; (†) Sheppard M. Practical Cardiovascular Pathology, 2nd edition: Taylor & Francis; 2011.
