# Supplementary material for: Autophagy and Inflammasome Activation in Dilated Cardiomyopathy
Source: J Clin Med. 2019 Sep 21;8(10):1519. doi: 10.3390/jcm8101519 (PMC6832472; doi:10.3390/jcm8101519)
Supplement: Supplementary file 1 [file jcm-08-01519-s001.zip › Supplementary Material/Suppl M&M.docx]

*Histology, histochemistry, immunohistochemistry and immunofluorescence assays.*

Gomori trichrome staining was performed for fibrosis assessment, while Congo red staining was employed to detect amyloid. Immunohistochemistry and immunofluorescence stainings were performed as indicated in supplementary table 1. Images were acquired employing either a transmitted light microscope (Leica DMD 108, Leica, Germany), a confocal microscope (Leica TCS-SP2 or Leica TCS-SP8) or an epifluorescence microscope (Leica DMI 6000B). Morphometric analyses were carried out employing ImageJ software (https://imagej.net/Welcome).

*NMR analysis*

Dry extracts were re-dissolved in D_2_O phosphate buffer, pH 7.4, and NMR spectra were acquired on a Bruker Avance 500 MHz spectrometer (Bruker BioSpin, Rheinstetten, Germany). A data matrix of 24 samples and 451 spectra buckets, or NMR features, was uploaded to the MetaboAnalyst 3.0 platform(1) for statistical analysis with R version 3.2.2 (http://www.R-project.org). Each bucket was treated as an independent variable of both control and DCM groups and analyzed with an unpaired *t*-test. Hierarchical clustering was performed with the “*hclust”* function of “*stat”* R package on normalized data after autoscale features standardization. Pearson's correlation was used for distance measure parameter and Ward's linkage as clustering algorithm. Concentration tables for 22 identified metabolites were uploaded on Metabo-Analyst 3.0 platform for Pathway Enrichment Analysis, and analyzed with the Global Test approach(2). Pathway Topology Analysis was performed by Relative Betweenness Centrality approach(3).

*Oxygen consumption rates*

OCR for the mitochondrial stress test was determined following the manufacturer’s instructions. For the statistical analyses, all the OCR values were normalized with those of one control CPC line, then the measurements of the four control CPC lines were averaged and the same was done with the four CPC lines obtained from the DCM patients.

*Realtime PCR analysis of miRNA expression*

miRNA reverse transcription was performed using the TaqMan MicroRNA Reverse Transcription Kit (Applied Biosystems), using specific primers. 1.3μL of the RT product were used for the Real-time qPCR assay, employing a master mix (TaqMan Universal PCR Master Mix, with no UNG, Applied Biosystems) and TaqMan probes to evaluate the expression profile of miR-22, miR-146a and miR-146b. miR-16 and miR-92 were employed as endogenous controls for cells and tissues, respectively. The amplification protocol was carried out using the LightCycler 480 (Roche) instrument.

miRNA inhibition and miRNA overexpression

CPc were transfected at 70% of confluence with either 50 nM anti-hsa-miR-22-3p or with 50 nM Negative Control#1 (mirVana miRNA Inhibitor 2.0, Life technologies) and with either 50 nM has-miR-22-3p or with 50 nM miRNA MIMIC 2.0 Negative Control #1 (mirVana miRNA mimic 2.0, Life technologies) employing lipofectamine diluted in Opti-MEM (Gibco), following the manufacturer’s instructions. 72 hours after transfection, cells were fixed with 4% paraformaldehyde.

1. Xia J, Sinelnikov IV, Han B, Wishart DS. MetaboAnalyst 3.0--making metabolomics more meaningful. Nucleic acids research 2015;43:W251-7.

2. Hendrickx DM, Hoefsloot HC, Hendriks MM, Canelas AB, Smilde AK. Global test for metabolic pathway differences between conditions. Anal Chim Acta 2012;719:8-15.

3. Aittokallio T, Schwikowski B. Graph-based methods for analysing networks in cell biology. Brief Bioinform 2006;7:243-55.
