# Supplementary figures and images for: Autophagy and Inflammasome Activation in Dilated Cardiomyopathy
Source: J Clin Med. 2019 Sep 21;8(10):1519. doi: 10.3390/jcm8101519 (PMC6832472; doi:10.3390/jcm8101519)

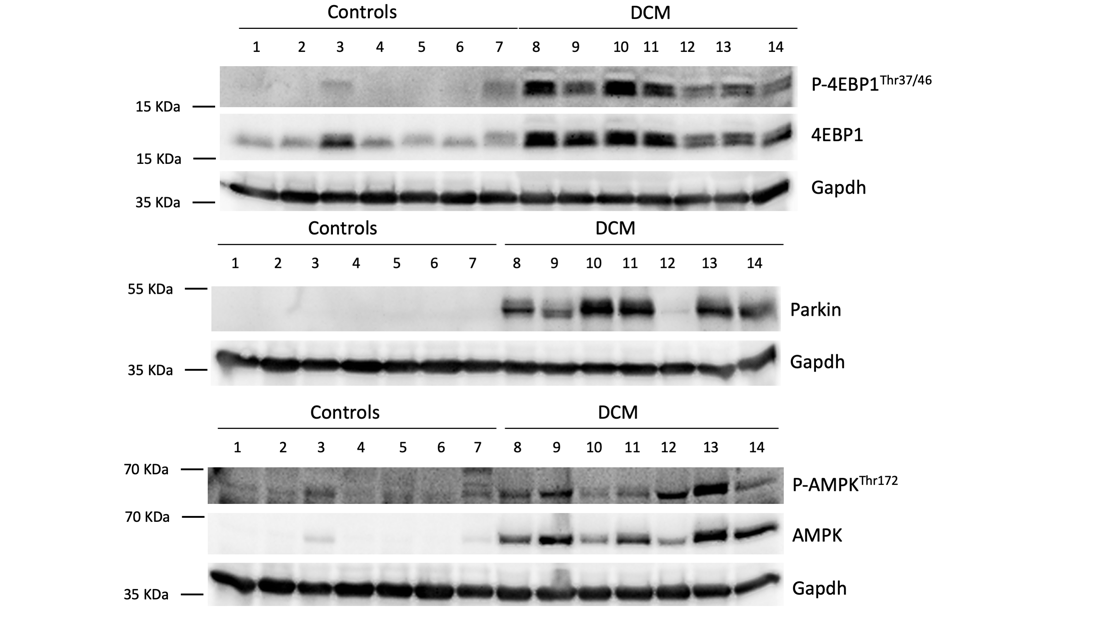

Supplement: Supplementary file 1 [file jcm-08-01519-s001.zip › Supplementary Material/Supp Fig 1.tif]

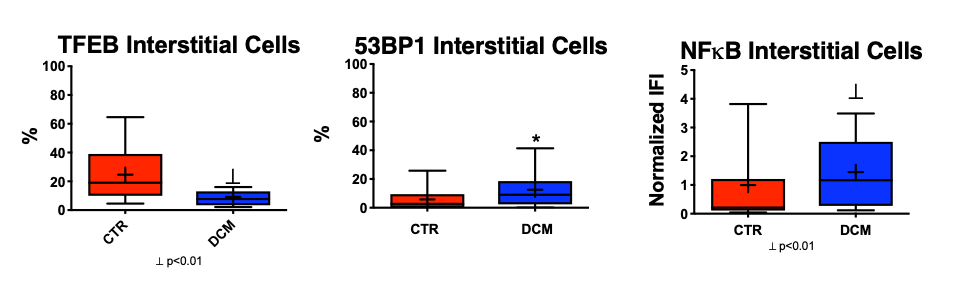

Supplement: Supplementary file 1 [file jcm-08-01519-s001.zip › Supplementary Material/Suppl Fig 2.tiff]

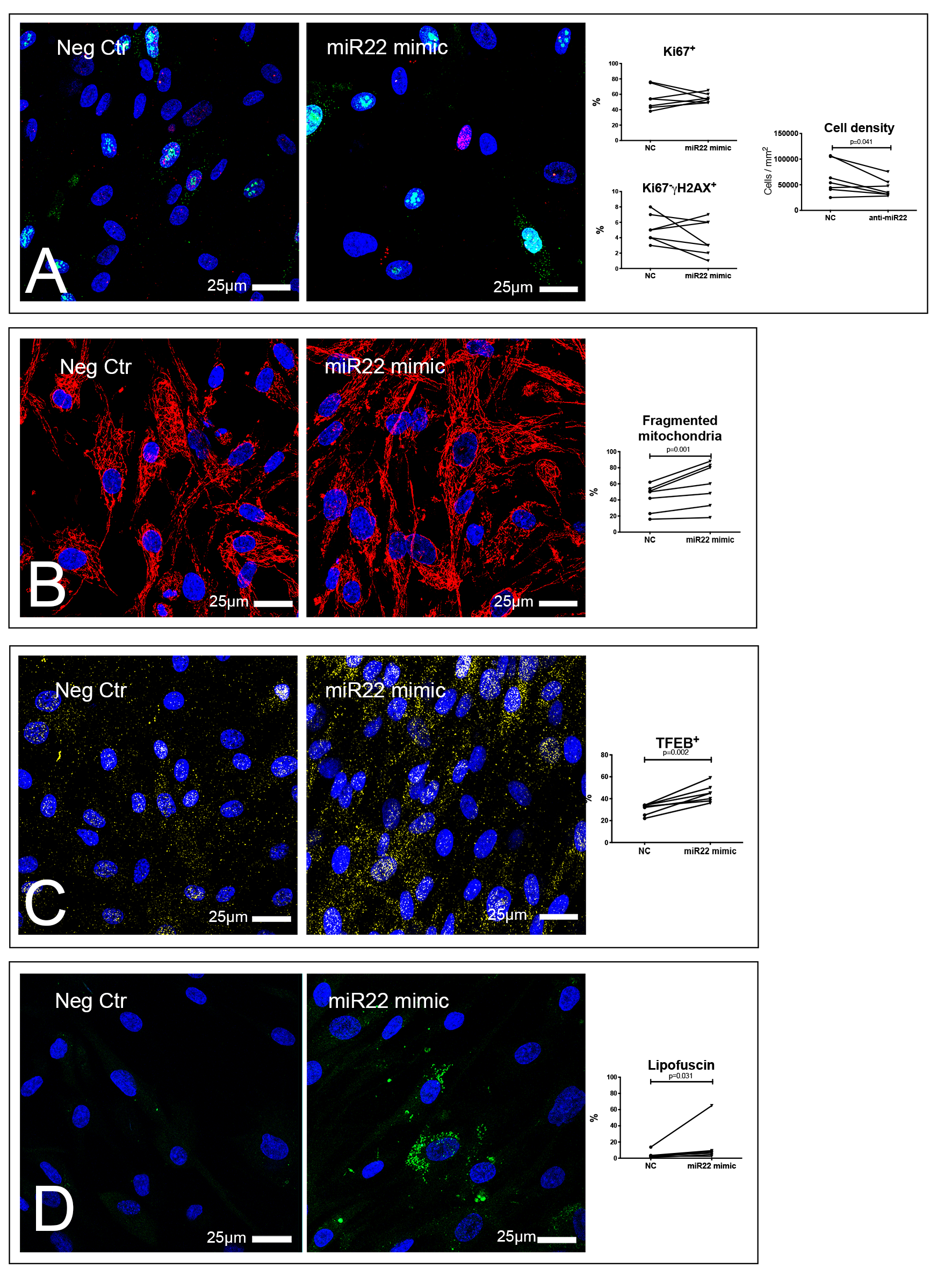

Supplement: Supplementary file 1 [file jcm-08-01519-s001.zip › Supplementary Material/Suppl Fig 3.tif]
